# A Computational Investigation on the Connection between Dynamics Properties of Ribosomal Proteins and Ribosome Assembly

**DOI:** 10.1371/journal.pcbi.1002530

**Published:** 2012-05-24

**Authors:** Brittany Burton, Michael T. Zimmermann, Robert L. Jernigan, Yongmei Wang

**Affiliations:** 1 Department of Chemistry, The University of Memphis, Memphis, Tennessee, United States of America; 2 Laurence H. Baker Center for Bioinformatics and Biological Statistics, Department of Biochemistry, Biophysics and Molecular Biology, Bioinformatics and Computational Biology Graduate Program, Iowa State University, Ames, Iowa, United States of America; Centro de Investigación Príncipe Felipe (CIPF), Spain

## Abstract

Assembly of the ribosome from its protein and RNA constituents has been studied extensively over the past 50 years, and experimental evidence suggests that prokaryotic ribosomal proteins undergo conformational changes during assembly. However, to date, no studies have attempted to elucidate these conformational changes. The present work utilizes computational methods to analyze protein dynamics and to investigate the linkage between dynamics and binding of these proteins during the assembly of the ribosome. Ribosomal proteins are known to be positively charged and we find the percentage of positive residues in r-proteins to be about twice that of the average protein: Lys+Arg is 18.7% for *E. coli* and 21.2% for *T. thermophilus*. Also, positive residues constitute a large proportion of RNA contacting residues: 39% for *E. coli* and 46% for *T. thermophilus*. This affirms the known importance of charge-charge interactions in the assembly of the ribosome. We studied the dynamics of three primary proteins from *E. coli* and *T. thermophilus* 30S subunits that bind early in the assembly (S15, S17, and S20) with atomic molecular dynamic simulations, followed by a study of all r-proteins using elastic network models. Molecular dynamics simulations show that solvent-exposed proteins (S15 and S17) tend to adopt more stable solution conformations than an RNA-embedded protein (S20). We also find protein residues that contact the 16S rRNA are generally more mobile in comparison with the other residues. This is because there is a larger proportion of contacting residues located in flexible loop regions. By the use of elastic network models, which are computationally more efficient, we show that this trend holds for most of the 30S r-proteins.

## Author Summary

Ribosomes are complex cellular machines that synthesize new proteins in the cell. The accurate and efficient assembly of ribosomal proteins (r-proteins) and ribosomal RNA (rRNA) to form a functional ribosome is important for cell growth, metabolic reactions, and other cellular processes. Additionally, some antibacterial drugs are believed to target the bacterial ribosome during its construction. Hence, ribosomal assembly has been an active research topic for many years because understanding the assembly mechanisms can provide insight into protein/RNA recognitions important in many other cellular processes, as well as optimize the development of antibacterial therapeutics. Experimental studies thus far have provided still limited understanding about the assembly process. To further understand the assembly process, we have computationally studied the dynamic properties that r-proteins exhibit during assembly and the relationship between dynamics, physical properties, and binding propensity. We observe significant charged interactions between r-proteins and rRNA. We also detect a strong correlation between contact residues and their dynamic mobilities. Protein residues contacting with rRNA are observed to be more mobile in comparison with other residues. We also relate the location of the r-protein in the fully assembled ribosome to its susceptibility for large conformational changes prior to binding.

## Introduction

Ribosomes are the macromolecular machines that synthesize proteins in all living organisms. They are composed of ribosomal RNA (rRNA) and ribosomal proteins (r-proteins) that self-assemble into functional units. The efficient and accurate self-assembly of the active ribosome in vivo is essential for cell growth because new ribosomes and proteins must be produced in order for cells to grow. It is estimated that approximately 60% of all cellular transcriptional activities have been attributed to the synthesis of rRNA in a rapidly growing cell [1] and 40% of the total energy of an *E. Coli* cell is directed towards the synthesis of proteins [2]. It is therefore not surprising that ribosome biogenesis in cells is intricately regulated. Elucidating this complex regulation network has become the focus of a rapidly developing field.

**Figure 1:**
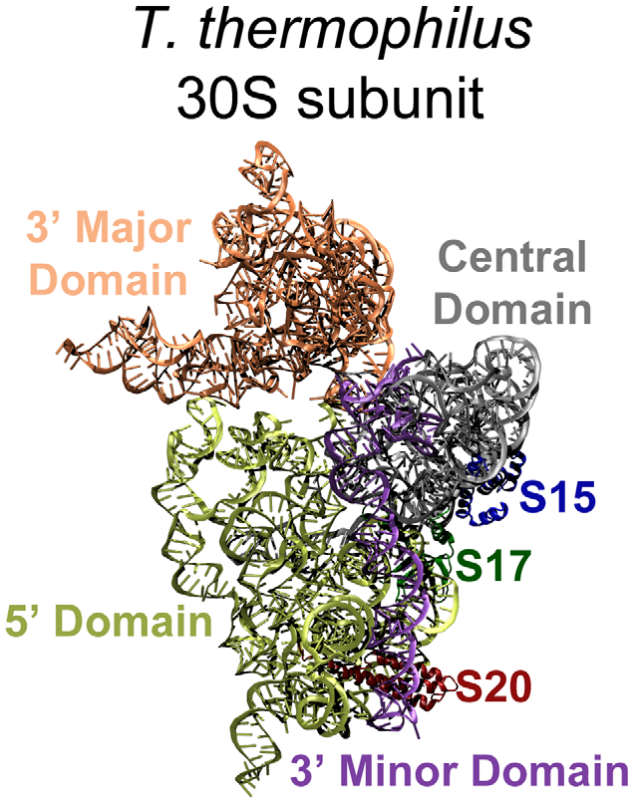
The 30S *T. thermophilus* subunit (1J5E), interface side. The 16S rRNA and r-proteins of interested are highlighted: 5′ Domain yellow, Central Domain grey, 3′ Major Domain orange, and 3′ Minor Domain purple; S15 blue, S17 dark green, and S20 dark red. The *E. coli* structure (2AVY) is nearly identical, but slight structural differences for the proteins of interest are discussed in the text and visualized in Figure 2. The remaining r-proteins have been removed for better visualization of the 16S rRNA domains.

The assembly of the ribosome requires the orchestration of highly coordinated events that involve both rRNA folding and r-protein binding. While many cofactors have been identified that participate in assembly *in vivo*, active functional units can be assembled *in vitro* in the absence of these cofactors [3]. The small 30S subunit of the bacterial ribosome (see Figure 1), which is composed of 16S rRNA and 21 r-proteins, has been more extensively studied than other structural assemblages and is a good system to analyze in order to determine what is important for the ribonucleic particle (RNP) assembly. In particular, the 30S subunit was the first to be reconstituted from purified components by the Nomura group in the late 1960's [4]. The reconstituted 30S active particles showed nearly the same activities in all performed biochemical assays. This ability to reconstitute active particles *in vitro* allows for in-depth exploration of the roles of the individual components in ribosome assembly and their functions by the combinatorial addition and omission of individual components [3], [5]–[6]. These experiments revealed that the 30S subunit assembles in a sequential and ordered process [3]. The Nomura group also provided a detailed assembly map describing the sequential and interdependent binding of all r-proteins [7]. The map also classified the proteins as primary, secondary, and tertiary binders, depending on their ability to bind to 16S rRNA. The primary proteins bind to bare rRNA, secondary proteins can bind to 16S rRNA after at least one primary protein has already bound, and tertiary proteins require at least one primary and one secondary protein [6].

The Nomura assembly map reflects the equilibrium thermodynamics of r-protein binding with 16S rRNA to intermediates. Using chemical probing methods, these binding kinetics were more recently studied by Powers *et al.* [8] Based on their experimental results, the r-proteins were divided into early, mid, mid-late and late binders. The kinetics data were partially in agreement with thermodynamic data in that the tertiary binding proteins were consistently found to be late binders. The availability of atomic structures of the 30S subunit [9]–[10] provided tremendous new opportunities to understand the assembly mechanism. Most of the knowledge gained in earlier experimental studies was found to be consistent with the determined structures.

In the meantime, significant progress was made with experimental methods to probe the ribosome assembly mechanism. Time-resolved X-ray-dependent hydroxyl radical footprinting [11]–[12] provides resolution on the order of milliseconds, much shorter than other chemical probing methods [8]. Directed hydroxyl radical probing [13]–[15] allows for the detection of specific interaction sites between proteins and RNA. The Williamson group used PC/QMS (pulse-chase followed with quantitative mass spectrometry) to measure the kinetics of individual protein binding during the assembly of the full ribosomal complex [16]. New experimental data suggest that ribosome assembly proceeds via multiple parallel pathways [16]–[17] rather than a single pathway involving the formation of a single rate-determining “reaction intermediate” RNP [18]. Current understanding of the ribosome assembly process suggests it is similar to protein folding in that it can proceed via multiple pathways across a rugged energy landscape.

Many computational studies have shed light on some important aspects of ribosome structure and function. Molecular dynamics simulations have been performed to analyze ribosome interactions with and the accommodation of transfer RNA (tRNA) during translation [19]–[22], as well as to characterize the interactions between cognate tRNA codons and their messenger RNA (mRNA) anticodons [23]–[24]. Other simulations and calculations used structures from various stages of translation to study the behavior of incoming mRNA transcripts [25] and nascent polypeptides in the ribosome's exit tunnel [26]–[27]. Interactions between ribosomes and members of a class of antibiotics called aminoglycosides have been elucidated via computational techniques [28]–[31] and have shed light on important interactions between these small molecules and the decoding center of the ribosome. Investigations of the interactions between the ribosome and important non-ribosomal proteins, such as the elongation factor EFTu, have been performed using MD [32] and quantum level calculations [33]. Other quantum calculations have been used to address the function of ribosome catalysis, such as the mechanism of and possible transition states in peptide bond synthesis [34]–[35]. These investigations have enriched the current understanding of ribosomal function and additional computational analyses on the dynamical structure of the ribosome and its components can further elucidate the mechanisms by which the ribosomal machinery assembles and operates.

Despite significant progress in recent years, the understanding of ribosome assembly remains limited. One major obstacle in this field is elucidating the mechanisms of coordinated RNA folding, protein binding, and the associated conformational changes of RNA and r-proteins [36]. Although earlier studies suggested [37] that r-proteins adopt the same structures in solution as in the assembled ribosome, more recent studies suggest [36] that there are conformational changes in the r-proteins and rRNA upon forming the complexes. Predicting RNA structure is also one of the most challenging topics in structural biology because a single stranded RNA can adopt a variety of secondary and tertiary structures. The 16S rRNA molecule in a ribosome is divided into four domains: the 5′ domain, the central domain, the 3′ major domain and the 3′ minor, each with a well-defined structure (see Figure 1). Magnesium ions are thought to stabilize the secondary structure of RNA and many r-proteins are thought to stabilize the tertiary structures. Many of the r-proteins interact with and bind to only one domain, but a few associate with more than one, such as S20 which interacts with both the 5′ and the 3′ minor domains. The Harvey group [38] analyzed the atomic contacts of r-proteins with RNA in the 30S subunit structure and reported the interesting observation that most of the late binding r-proteins were found to bind at the 3′ end of 16S RNA. This observation was consistent with the earlier understanding that 16S RNA folds with 5′ to 3′ polarity [6], [14]. The Harvey group further used coarse-grained representations of RNP structures to examine the potential fluctuations of binding sites when proteins were removed or added. Their study shows that the binding sites of primary proteins are formed first and, once associated, these proteins help organize the late binding sites. Trylska *et al.* [39] calculated the binding energy of individual r-proteins with the 16S RNA by solving the Poisson-Boltzmann equation, which accounts for electrostatic interactions. Though the calculated binding energies varied, some late binders were found to have less favorable binding free energies while the early binders were found to be more favorable, an observation consistent with known experimental results. Other studies used various coarse-grained representations to explore the global motions of the ribosome [25], [40]–[43] and the assembly of the 30S [44]–[45]. Despite the coarse representations of ribosomal structure, some of the known dependencies of r-protein and rRNA binding were captured in these computational studies.

Ribosome assembly remains an active research field. A better understanding of its assembly mechanisms will provide valuable biochemical insight into cellular regulation and will allow for the optimal development of ribosome-targeted drugs. While experimental studies continue to make great progress, computational studies reported so far are still limited. Most of the earlier reported computational studies have used coarse-grained representations of the ribosome. To truly understand the specific binding of r-proteins with 16S RNA, atomistic details need to be considered. Because assembly involves both RNA folding and protein binding, the examination of individual components before and after binding in atomistic detail is necessary. Here we specifically investigate the potential correlation between r-protein dynamics properties and their binding properties. The aim is to answer the following specific questions: what are the key residues that bind to the 16S rRNA? Are these key residues more flexible than the others? Do free r-proteins adopt the same conformations as those found in the assembled 30S subunit? To explore the answers to these questions, we rely on the use of atomistic molecular dynamic simulations of r-proteins as well as other methods developed in our own group.

## Results/Discussion

### Ribosomal proteins are enriched with positively charged amino acids

Ribosomal proteins are known to be positively charged and many of these positively charged amino acids, especially those residues on the long extension tails, were found to interact with RNA [10], [46]–[47]. We performed a simple calculation of the net charge of ribosomal proteins based on the sequences reported for the 2AVY and 1J5E structures, counting Asp and Glu as −1, Lys and Arg as +1, with all other residues treated as neutral. Of course, some of these residues might have some charge because of shifted p*K_a_* values due to their location in the tertiary structure, but we will ignore these minor effects at present. Table 1 presents the net charge of r-proteins for the two species. The two r-proteins that are not positively charged could be explained by their special positions in the assembly map: S2 is the last protein to assemble [7] and S6 is known to form a dimer with S18 [48]–[49], which is positively charged, before associating with rRNA. The remaining r-proteins are all positively charged. We also note that the charge on r-proteins from *T. thermophilus* is on average higher than that for the *E. coli* proteins, which may relate to the general observation that ribosomal subunits for thermophiles such as *T. thermophilus* are more stable than those of mesophiles such as *E. coli* [50]. Moreover, ribosomal proteins are enriched with positively charged amino acids. The typical percent of amino acids for Lys, Arg, Glu and Asp are 5% each for cytosolic proteins [51]. However, in the case of r-proteins, the total percentage of Lys and Arg is approximately 20% (18.7% for *E. coli* and 21.2% for *T. thermophilus*), while the sum of Glu and Asp percentages remained near 10%. Klein et al had earlier examined the amino acid distributions of r-proteins in the large subunit (50S) and reported a similar bias toward the positively charged amino acids [46].

**Table 1:** Net charges of r-proteins.

r-protein	*E. coli*	*T. thermophilus*
**S2**	−1	−7
**S3**	19	21
**S4**	17	23
**S5**	9	7
**S6**	−12	0
**S7**	14	15
**S8**	5	12
**S9**	16	16
**S10**	3	10
**S11**	15	16
**S12**	21	27
**S13**	14	20
**S14**	15	16
**S15**	8	8
**S16**	6	11
**S17**	6	15
**S18**	12	19
**S19**	12	10
**S20**	16	25
**S21**	14	12

Note: S21 for *T. Thermophilus* is called THX.

We have further examined the contacts made between r-proteins and the RNA based on the atomic structures of the 30S subunit from the two species. Here, a contact is defined as having any atoms of a protein residue within 3.5 Å of any rRNA nucleotide atoms. Table 2 presents the number of contacts made by each r-protein, along with the number of contacts with positively charged residues. It is clear that a high percentage of contacts between r-proteins and rRNA are made by positively charged residues. The total average percentages of contacts made by positively charged residues are 39% for *E. coli* and 46% for *T. thermophilus*, and both are significantly higher than the total percentage of the positively charged amino acids in r-proteins for the two species. These results together affirm the known importance of charge-charge interactions in the ribosome [10], [46]–[47].

**Table 2:** Contacts between r-proteins and r-RNA in total and for charged residues.

	*E. Coli* Contacts (3.5 Å cut off)				*T. Thermophilus* Contacts (3.5 Å cut off)			
r-proteins	Total	Pos.	Neg.	% Pos.	Total	Pos.	Neg.	% Pos.
**S2**	19	7	0	37%	17	5	1	29%
**S3**	40	7	2	18%	42	13	2	31%
**S4**	64	23	3	36%	83	38	4	46%
**S5**	46	13	0	28%	48	19	1	40%
**S6**	8	3	0	38%	14	8	1	57%
**S7**	29	15	3	52%	49	30	2	61%
**S8**	37	10	3	27%	40	12	2	30%
**S9**	81	44	1	54%	88	45	5	51%
**S10**	42	15	1	36%	49	17	2	35%
**S11**	52	19	0	37%	50	16	0	32%
**S12**	75	28	4	37%	83	44	5	53%
**S13**	48	22	0	46%	71	35	0	49%
**S14**	54	23	0	43%	53	29	3	55%
**S15**	42	8	3	19%	43	15	3	35%
**S16**	42	20	3	48%	57	29	2	51%
**S17**	32	14	2	44%	70	33	2	47%
**S18**	30	16	0	53%	18	13	0	72%
**S19**	37	17	1	46%	49	22	1	45%
**S20**	52	24	2	46%	62	32	4	52%
**S21**	6	2	3	33%	30	16	1	53%
**Total**	**836**	**330**	**31**	**39%**	**1016**	**471**	**41**	**46%**

Note: The total number of protein contacts for S15, S17, and S20 above differs from the total number of contact residues presented in Supplementary Tables S1, S2, S3 because some protein residues are in contact with more than one nucleotide, which are presented here as multiple contacts.

### Structures and contact residues are more conserved than sequences

**Figure 2:**
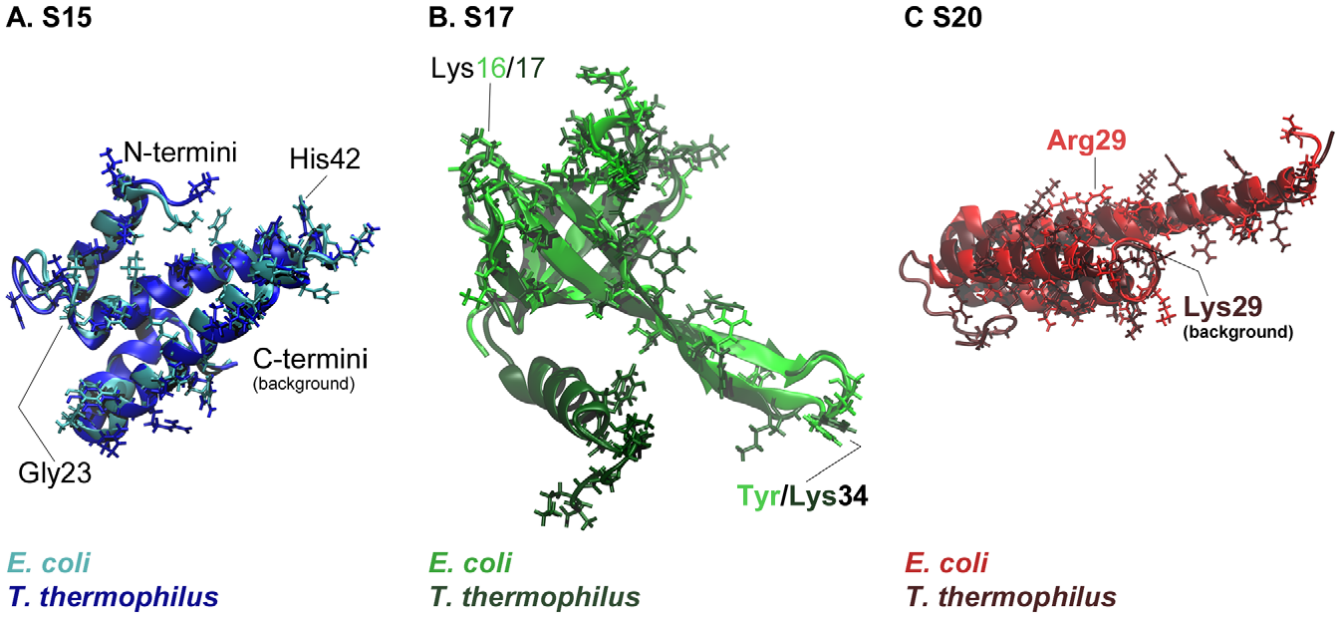
Comparisons of S15, S17, and S20 proteins from two different species. *E. coli* proteins are shown in the lighter shade and *T. thermophilus* in the darker shade. Contact residues are shown as stick representations and some important parts of the proteins, discussed in the text, are labeled.

Figure 2 shows structural alignments for the three proteins from the two species. The percentages of sequence identity between the two species are 60% for S15, ∼40% for S17, and ∼28% for S20, but the percentages of conserved residue class are considerably higher: 75% for S15, ∼58% for S17, and ∼47% for S20. Thus, the structures for the three ribosomal proteins are well conserved, with RMSD values of 1.1 Å for S15, 1.4 Å for S17, and 2.1 Å for S20. In the cases of S17 and S20 from *T. thermophilus*, there are extra C-terminal regions, as shown in Figures 2b and 2c.

Residues that contact rRNA exhibit higher than average sequence conservation. For S15, the percent of conserved contact residues is about 54% (52% for *E. coli* and 56% for *T. thermophilus*), which is just under the overall sequence conservation. For S20, the percentage of conserved contact residues is 38% for *E. coli* and 35% for *T. thermophilus*, both of which are considerably higher than the overall sequence conservation. For S17, the percentage of conserved *E. coli* contacting residues (52%) is higher than the overall sequence conservation, whereas that for *T. thermophilus* contacting residues (31%) is less. The conserved contact residues percentages for S17 and S20 from *T. thermophilus* are lower than those for *E. coli* because *T. thermophilus* has extra C-terminal regions that make several additional non-conserved contacts. (Supplementary Tables S1, S2, S3 present the contact residues for S15, S17 and S20 for the two species, with conserved residue identities in red and conserved side chain types, largely Lys/Arg substitutions, colored green.)

Further analysis of the identities of these contact residues reveals that, aside from the positively charged residues, His, Thr, Ser, and Gln are also common, all of which are polar and can form hydrogen bonds with rRNA. For example, of the twenty-seven *E. coli* S15 contacts, five are basic (Lys48, Arg54, Arg64, Lys65, and Lys73), five are histidines (His38, His42, His46, His50, and His51), ten are polar (Ser2, Thr5, Thr8, Thr22, Ser24, Gln28, Gln35, Ser52, Ser61, and Gln62), and one is aromatic and polar (Tyr69). The remaining six contacts are acidic (Asp21 and Asp49) or nonpolar (Gly23, Leu31, Leu39, and Gly55). Therefore, most contacts between the r-proteins and the rRNA are either charged interactions, or hydrogen bonds, with few aromatic stacking or nonpolar interactions.

### Dynamics and conformational changes of S15

S15 is a primary binding protein which binds in the 3′ major domain of 16S RNA. In the assembled 30S subunit, S15 is solvent-exposed and located on the back of the 30S subunit body. The 16S RNA binding site of S15 is at the three-way junction of helices 20, 21, and 22 in the 16S central domain. The primary, secondary, and tertiary structures of S15 are highly conserved across species: four bundled α-helices are connected by short loops (Figure 2a). All 16S rRNA contact residues are found on one side of S15, located on helices 1, 2 and 3 and the loops connecting the three helices, but helix 4 does not have any contacts with rRNA.

In previous structural studies, X-ray [52]–[54] and NMR [55]–[56] derived structures were reported and the only significantly different conformation reported was in the crystal structure [52] where helix 1 was rotated 90° away from the remaining bundled helices. Additional studies have been published about the role of S15 in ribosome assembly and antibiotic responses with mutagenesis studies [57] and MD simulations, studying the effects of Mg^2+^ ions on the protein alone and with its rRNA binding site [56]. It has been suggested that this protein acts as a bridge between the large and small subunits in the fully assembled ribosome [58].

**Figure 3:**
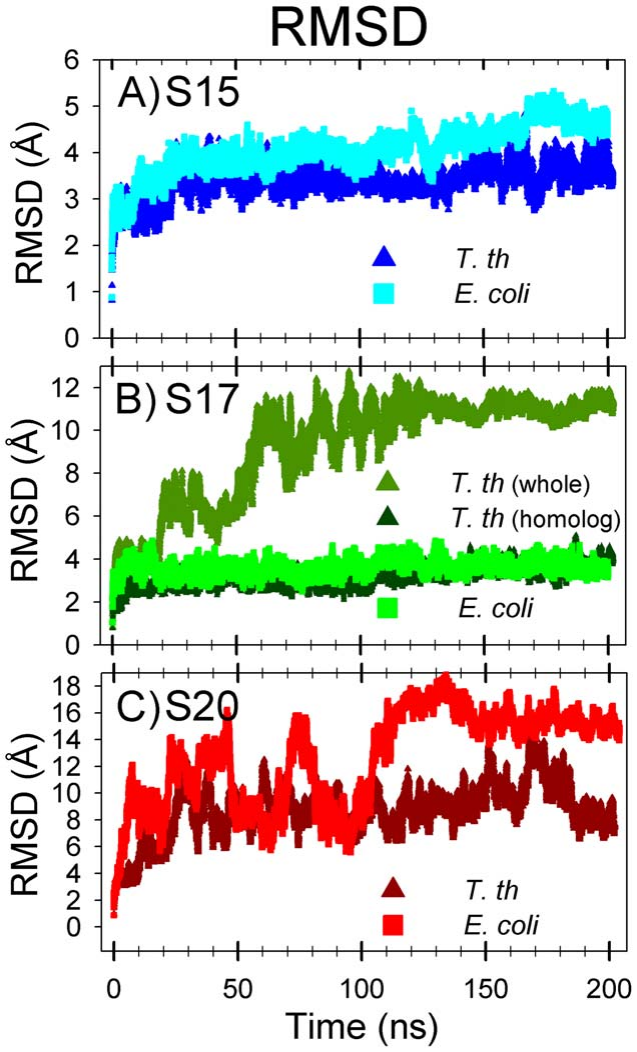
RMSD values for S15, S17, and S20 proteins. *E. coli* proteins are represented by lighter squares and *T. thermophilus* by darker triangles. The S17 include the RMSD value for just the part of the structure that is homologous (dark green) to *E. coli* S17 (omitting the extra *T. thermophilus* C-terminal part). Notably, this C-terminal part of S17 causes the *T. thermophilus* to greatly increase its overall mobility.

**Figure 4:**
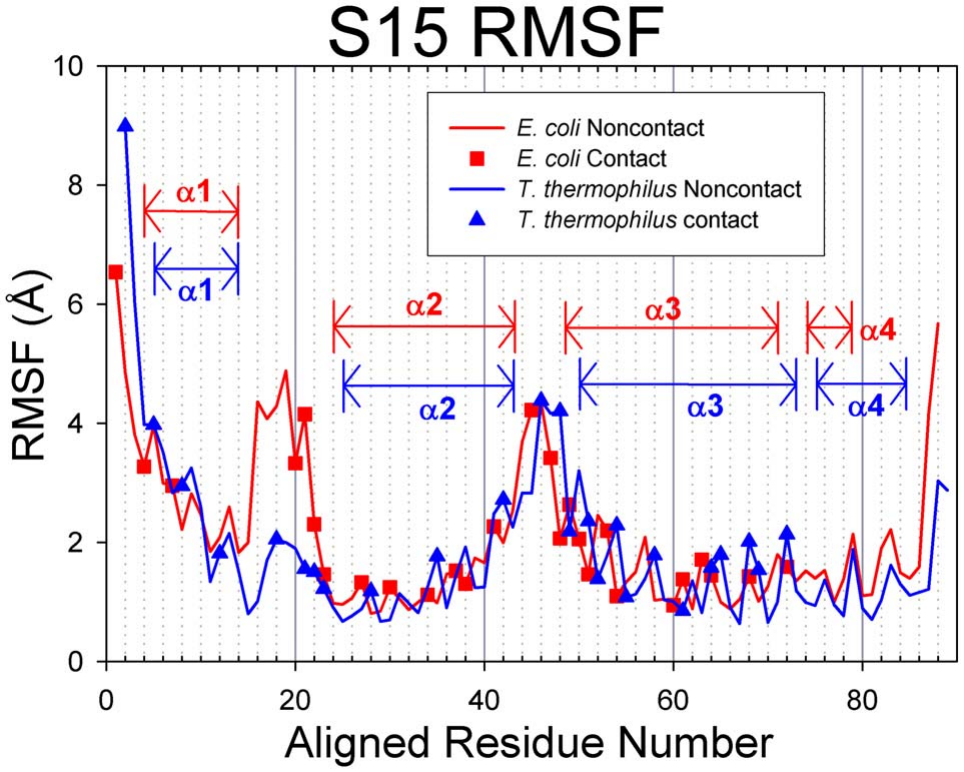
RMSF values for S15. *E. coli* proteins are represented in red with squares indicating contact residues and *T. thermophilus* proteins are colored blue with triangles for contacts. In these figures, the proteins have been sequentially aligned to demonstrate the behaviors of the conserved structural elements. Aligned Residue Numbers, therefore, do not necessarily reflect the actual residue indices of the protein sequence.

**Figure 5:**
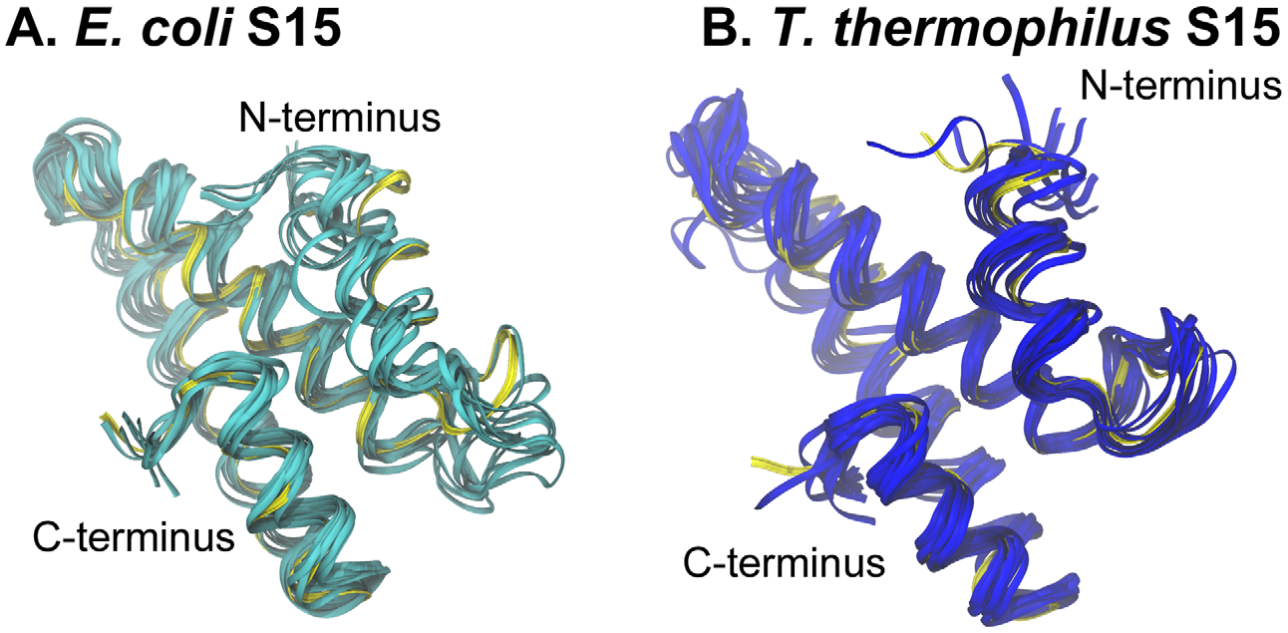
S15 structural variations during MD simulation. Backbone snapshots of both proteins are in shades of blue (*E. coli* light blue; *T. thermophilus* dark blue). Backbone starting structures are shown in yellow.

Root-mean-square deviations (RMSD) were calculated from the molecular dynamics simulations of the S15 protein and are presented in Figure 3a. The S15 from the two species exhibit relatively low RMSD values during MD simulations, with values remaining below 5 Å. Figure 4 presents the root-mean-square fluctuation (RMSF) values calculated over the period of time from 10 ns until the end of the simulation. Contact residues are shown as solid symbols in the plot. High RMSF values were observed for the loop connecting helices 2 and 3, and several conserved contact residues are located in this loop. The contact residues found on helices 2 and 3 have very low RMSF values, whereas helix 1 and the loop connecting helices 1 and 2 have a few contact residues with moderate RMSF values. Helix 4, which has no contacts with 16S RNA retains its helical structure during the MD simulation and has moderate RMSF values. Representative backbone structures for *E. coli* and *T. thermophilus* S15 are depicted in Figure 5. The proteins retain their secondary and tertiary structures during the MD simulations and only small conformational changes are observed for either S15 protein. This indicates that the S15 protein from both organisms is a relatively stable protein in solution and that the conformations observed during the simulations are similar to that of the attached protein in the assembled ribosome.

Table 3 compares the average RMSF for contact residues with respect to average RMSF for all residues. The average RMSF value for all *E. coli* S15 residues is 2.11 Å and for all contact residues is 2.24 Å. For *T. thermophilus* S15, all residues average RMSF is 1.84 Å and all contacts is 2.37 Å. These differences are small, but statistical analysis shows that S15 contact residues are positively enriched with mobile residues, as indicated by enrichment factors greater than 1 for both species (Table 3; EF = 1.08 and p-value = 0.217 for *E. coli*; EF = 1.46 and p-value = 0.008 for *T. thermophilus*, see Methodology for explanation of enrichment factors and the p-value). The P-values for these enrichment factors signify that the mobility enrichment of *T. thermophilus* contact residues is significant while it may not for *E. coli*.

**Table 3:** Average MD RMSF values (in Å; standard deviations in parentheses) and enrichment factors EF.

		All Residues		All Contacts		Contact EF	P-value
***E. coli***	**S15**	2.11	(1.24)	2.24	(1.27)	1.08	0.217
	**S17**	1.85	(0.94)	2.28	(0.92)	1.10	0.199
	**S20**	8.82	(3.17)	9.14	(3.34)	1.06	0.215
***T. thermophilus***	**S15**	1.84	(1.29)	2.37	(1.66)	1.46	0.008
	**S17**	4.68	(2.93)	5.74	(3.40)	1.40	0.008
	**S20**	6.96	(2.94)	7.62	(2.80)	1.15	0.057

### Dynamics and conformational changes of S17

In the 30S subunit, S17 is also solvent exposed and is located near S15 in the 5′ domain of the 16S rRNA. To date, no X-ray crystal structures have been determined for S17 alone, but a low resolution NMR solution structure has been presented for *Bacillus stearothermophilus* S17 [59]. The S17 structure found in the *E. coli* 30S subunit is comprised of a small β-barrel and an extended ß-hairpin loop (Figure 2b). The contact residues are located on one end of the β-barrel and in the extended ß-hairpin loop. The S17 from *T. thermophilus* has an extra C-terminal α-helix which makes additional contacts with the 16S rRNA (Figure 2b). Thus, *E. coli* contact residues exhibit somewhat higher conservation than the overall sequence does, whereas *T. thermophilus* contact residues are slightly less conserved than the sequence of the full-length proteins. In the *E. coli* 30S subunit, the S17 ß-hairpin loop is embedded in rRNA and contains five contacts, three of which are found contacting helix 11 of the central domain with two contacting the 5′ domain at helix 21. The axis of the β-barrel is oriented into the main part of the rRNA, and the end of the barrel nearest the RNA contains the remaining contact points, all of which contact the 5′ domain of 16S rRNA along helices 7, 9, and 11. Because these contacting residues associate with both the 5′ domain and the central domain, *E. coli* S17 is a plausible anchor between them. The *T. thermophilus* S17 also contacts these two 16S domains but includes an additional ten protein contacting residues in its C-terminal α-helix and coiled tail. These residues have a larger extent of contact with helix 11 and strengthen the association with the central domain at helices 20 and 27. Research indicates that the 30S subunit assembly begins at the 16S rRNA 5′ end [8] and, S17 appears to organize the 5′ region [14], so it is clear that the cooperative conformational changes and rRNA binding of this protein are likely to play an important role in the early stages of ribosome formation.

During the MD simulation of *E. coli* S17, the β-sheet structures remained stable: the average RMSD for this protein was relatively low (below 5 Å; lime green plot, Figure 3b). Conversely, a much higher RMSD was observed for S17 from *T. thermophilus* (olive green plot, Figure 3b), although the protein did take on a relatively stable conformation after ∼80 ns of simulation. Further investigation reveals that the extra α-helix in *T. thermophilus* S17 is responsible for the high RMSD values. The structurally homologous portions of the proteins have comparable RMSD values (*T. thermophilus* homolog: dark green plot, Figure 3b), both around 4 Å. The backbones of structurally homologous portions both retain their overall shape during the MD simulations.

**Figure 6:**
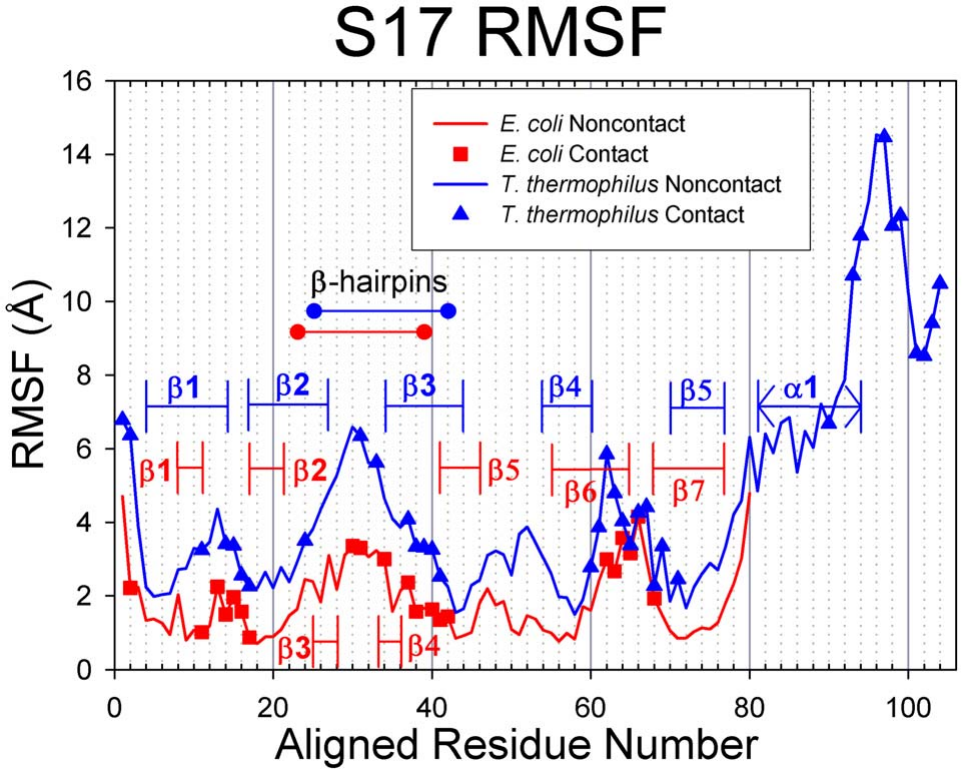
RMSF values for S17. *E. coli* proteins are represented in red with squares indicating contact residues and *T. thermophilus* proteins are colored blue with triangles for contacts. In these figures, the proteins have been sequentially aligned to demonstrate the behaviors of the conserved structural elements. Aligned Residue Numbers, therefore, do not necessarily reflect the actual residue indices of the protein sequence.

S17 RMSF values (Figure 6) were calculated from the MD simulations starting from the 10 ns point until the end of the trajectory. While the *T. thermophilus* S17 generally exhibited larger deviations from its starting structure than did the *E. coli* S17, when sequentially aligned, the RMSF values for the structurally homologous portions of the proteins correlate well. For *E. coli* S17, the loops connecting the ß-strands, the extended ß-hairpin loop, and both termini exhibit comparably high RMSF values, whereas the ß-strands participating in the ß-barrel (valleys in Figure 6) have low RMSF values. The same pattern is true for the homologous portion of the *T. thermophilus* RMSF plot, and the extra C-terminal region exhibits very large RMSF values. The contact residues in the *E. coli* S17 are located in the highly mobile ß-hairpin, the moderately mobile Loops 1 and 6, as well as the least mobile ß-strands of ß-barrel: ß5, the last residue of ß1, and the first of ß2. In *T. thermophilus* S17, there are four regions of the protein with high RMSF (the N-terminus, the ß-hairpin loop, Loop 4, and the C-terminus), all of which contain contact residues. In fact, every residue in Loop 4 is a contact residue, and residues close to each end of the loop also have high RMSF values. The three contact residues in the α-helix have high RMSF and the ten residues in the C-terminal coil have some of the highest RMSF, seven of which are contact residues. The low and moderate contact residues are found in the ß-barrel: ß1, Loop 1, ß2, and ß3.

**Figure 7:**
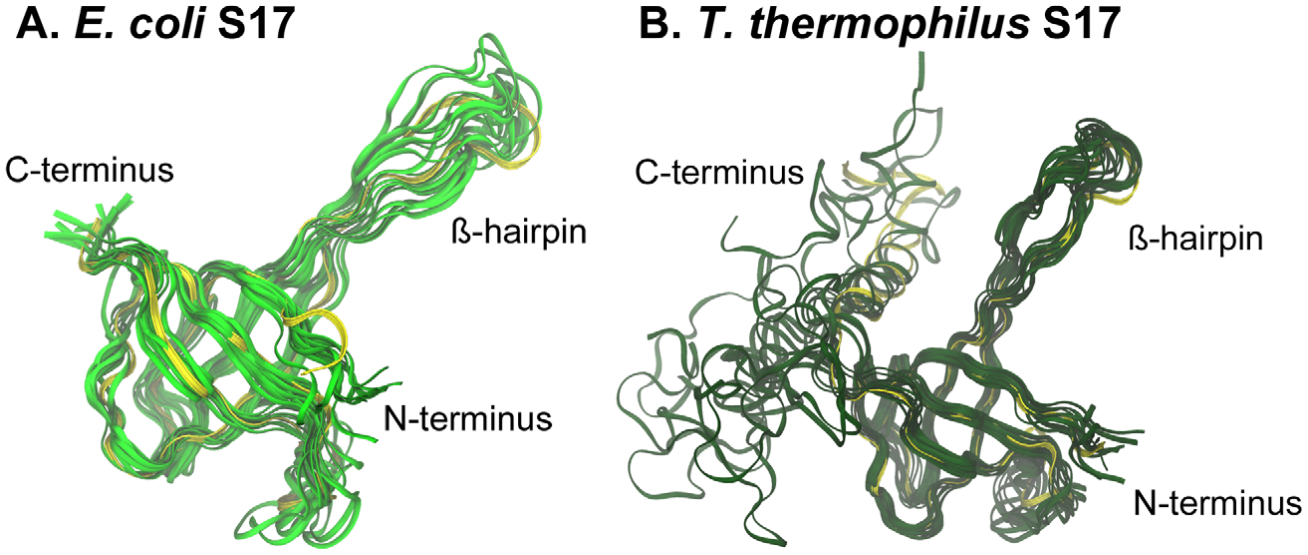
S17 structural variations during MD simulation. Backbone snapshots of both proteins are in shades of green (*E. coli* light green; *T. thermophilus* dark green). Backbone starting structures are shown in yellow.

Representative structures seen throughout the *E. coli* and *T. thermophilus* S17 simulations are shown in Figure 7. The RMSF data and these images indicate that the structurally homologous regions of the S17 protein behave similarly in solution and that the ß structures of both homologs retain their overall shape throughout the simulations, whereas the flexible C-terminal α-helix in *T. thermophilus* loses its helical structure. These data imply that the ß-barrel confers good stability in solution for the two species.

Further analyses of the relative mobility of contact residues shows similar trends as S15. The average RMSF (Table 3) for all residues in *E. coli* S17 is 1.85 Å and 2.28 Å for all contacting residues; for *T. thermophilus*, the average for all residues is 4.68 Å, and 5.74 Å for all contacting residues. The differences in these values, while small, indicate that contact residues are, on average, more mobile than all residues for both S17 proteins. Enrichment factors for S17 show positive mobility enrichment for contact residues in both species (Table 3; EF = 1.10 with p = 0.199 for *E. coli*; EF = 1.40 with p = 0.008 for *T. thermophilus*), with p-values indicating that *T. thermophilus* enrichment is significant while it may not be for *E. coli*.

### Dynamics and conformational change of S20

In the 30S subunit crystal structures from both species, protein S20 is found deeply embedded in the 16S rRNA. This protein contacts 16S RNA helices 6–9, 11, and13 in the 5′ domain and is the only r-protein to contact helix 44 in the 3′ domain. The structure of S20 consists of a unique set of three bundled α-helices, with helix 1 twice as long as the others, the N-terminus most deeply inserted into the subunit, and only a small portion of the three-helix bundle exposed to solvent. While the *E. coli* and *T. thermophilus* S20 proteins have a generally conserved tertiary body (Figure 2c), the *T. thermophilus* S20 crystal structure is missing its first seven residues and has an additional 15 residue C-terminal tail which the *E. coli* protein does not have.

**Figure 8:**
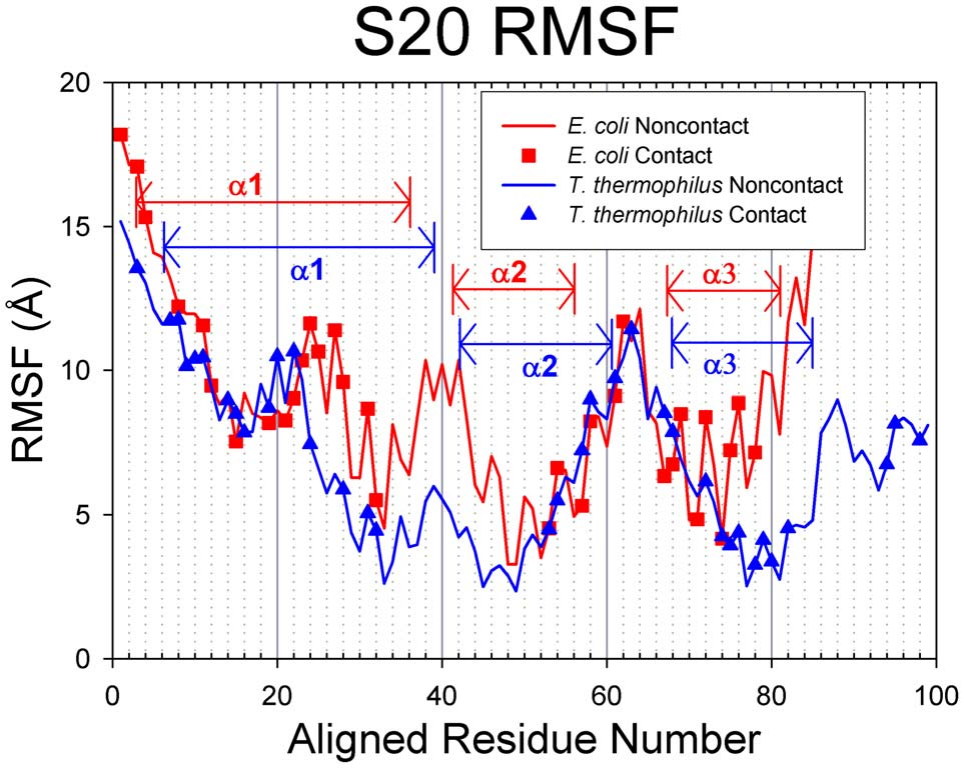
RMSF values for S20. *E. coli* proteins are represented in red with squares indicating contact residues and *T. thermophilus* proteins are colored blue with triangles for contacts. In these figures, the proteins have been sequentially aligned to demonstrate the behaviors of the conserved structural elements. Aligned Residue Numbers, therefore, do not necessarily reflect the actual residue indices of the protein sequence.

**Figure 9:**
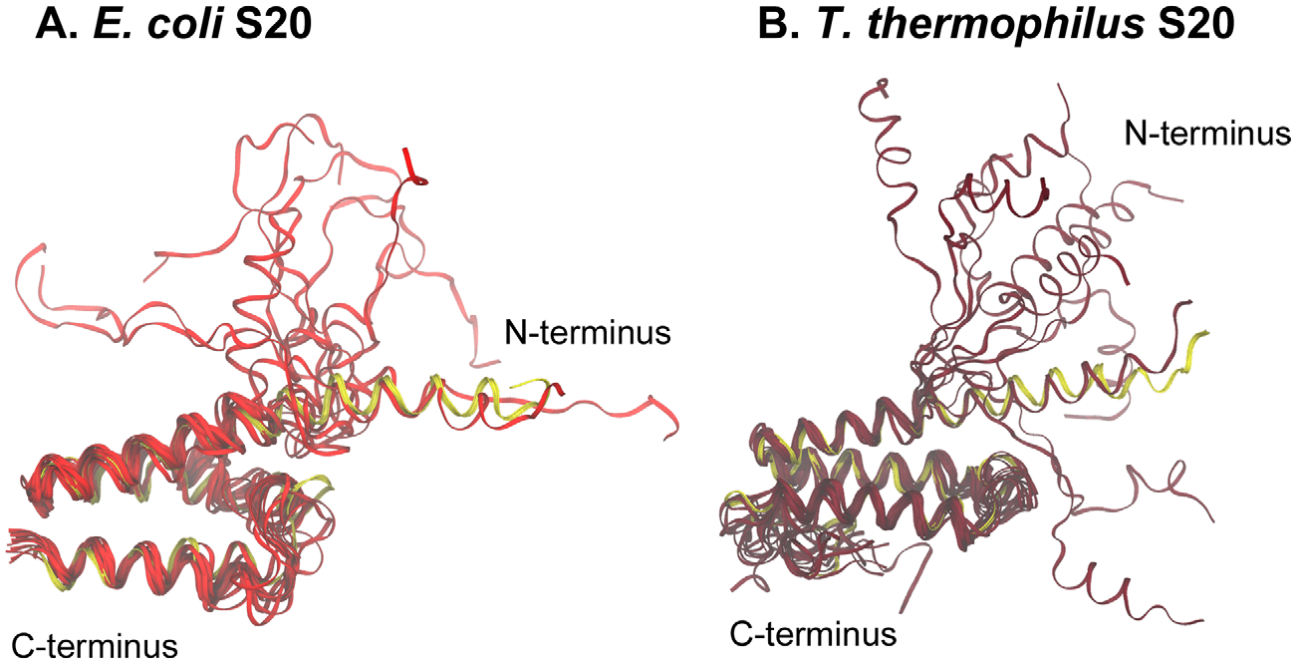
S20 structural variations during MD simulation. Backbone snapshots of both proteins are shown in shades of red (*E. coli* light red; *T. thermophilus* dark red). Backbone starting structures are in yellow.

The simulation RMSD values for S20 from both species oscillate wildly (Figure 3c), indicating the proteins conformation vary broadly from their starting conformations (up to ∼20 Å). Multiple length simulations (at least 200 ns) show that while S20 RMSD may remain within a range of 5–10 Å for a time, the protein does not adopt a solution-stable conformation. The S20 RMSF plots (Figure 8) have similar trends for both *E. coli* and *T. thermophilus* S20 proteins, and aside from the first portion of α1, the three α-helices are primarily located at valleys in the plots. The highly flexible region of α1 binds to rRNA helices 6, 7, and 13, whereas the nearby, more stable contact residues in α1 contact the tip of rRNA helix 44, a helix that has no contacts with any other small subunit proteins. The remaining contacts have relatively moderate or low RMSF values. As seen in the other proteins, the loop regions between the stable secondary structures are located at peaks in the RMSF plot, whereas the α-helical regions themselves correspond to the RMSF valleys. Visual inspection of the trajectories suggests that the major contributor to S20 flexibility is helix 1 (Figure 9), which extends deeply into the rRNA. The N-terminal portion of helix 1 bends and swings wildly during the MD simulations. *E. coli* helix 1 bends near Arg24 and Thr30 and *T. thermophilus* near Lys29.

Previous studies [60] have shown that the free S20 protein in solution does not exhibit the high percentage of α-helical regions as seen in the crystallized structure. The conformational variation exhibited by S20 in the work here is consistent with this data, and this flexibility coupled with the deep insertion of the protein into the folds of RNA in the fully-assembled ribosome indicate that S20 is stabilized primarily by its large number of contacts with the RNA.

The average RMSF trends (Table 3) for S20 contact residues are generally in agreement with the results presented for S15 and S17. For *E. coli*, the average RMSF for all residues is 8.82 Å and for all contact residues is 9.14 Å. In *T. thermophilus*, the average value for all residues is 6.96 Å and 7.62 Å for all contact residues. These data show that the mean RMSF for all contacts is greater than that for the whole structure, consistent with the results for S15 and S17. Both *E. coli* and *T. thermophilus* S20 proteins show positive enrichment of mobility in their contact residues (Table 3; EF = 1.06 with p-value = 0.215 for *E. coli*; EF = 1.15 with p-value = 0.057 for *T. thermophilus*). However, in this case, the p-values are both greater than 0.05, a typical threshold used for statistical significance test.

### General trends based on Elastic Network Modeling

To rapidly assess the potential connection between contacting residues and their mobilities, we use elastic network modeling which compute RMSF values using only a fraction of the computational resources required for the MD simulations. The elastic network models have been applied previously to the ribosome by us [25], [40], [45], [61], and in general the dynamics calculated via the Anisotropic Network Model [62]–[63] correlate reasonably well with those from the MD simulations. For example, the correlation coefficient between RMSF values calculated for *E. coli* S15 is 0.57, for S17 is 0.63, and for S20 is 0.81. ANM and MD predict similar patterns of mobility and stability, with most of the discrepancy at the terminal residues and highly flexible regions (such as S20 α-helix 1 and S17 ß-hairpin loop). In fact, if the first two and last two residues of *E. coli* S15 are excluded, the correlation factor increases to 0.67. The MD simulations typically predict greater terminal residue mobility (except for the highly mobile S20 helix 1) and the ANM calculations consistently predict higher fluctuation values for extended residues in the middle of the protein.

ANM mobility enrichment was calculated for all 19 r-proteins in the two 30S X-ray structures and results are presented in Table 4. Most r-proteins are significantly enriched for mobile residues at the rRNA contact points at the 0.05 level. Contacting residues are not only enriched, but they make up a subset of residues that is near maximal enrichment, for a given structure. Proteins S2, S6, S8, S18 and S19 do not show statistically significant enrichments and are colored red in Table 4. As mentioned earlier, S2 and S6 differ from the rest of r-proteins in that they do not have a net positive charge. Also S6 and S18 are known to form dimers in solution. Hence calculation of their dynamics as monomers may not reflect their true dynamics in solution. S8 is one of the primary binding r-proteins and S19 is one of the secondary binding r-proteins. At present, we do not know specific properties that may make these two proteins differ from the rest. Although their EF values are greater than one (rRNA contacts are more mobile), their p-values do not reach the level of high statistical significance (they are not a maximally enriched subset). In addition to those r-proteins, S14, S17 and S20 are not significantly enriched with mobile residues for *E. Coli*, but are statistically significant enriched for *T. Thermophilus*. On average, *T. thermophilus* proteins show a slightly increased enrichment relative to *E. coli*; with average enrichment factors of 1.51 and 1.46, respectively, with medians of 1.43 and 1.33. Of the 6 proteins categorized as being early by the Harvey group [38], two *E. coli* and five *T. thermophilus* have mobility enrichments significant at the 0.05 level. Of the six primary proteins identified by Nomura [7], three *E. coli* and five *T. thermophilus* are significant at the 0.05 level. Proteins involved later in assembly are not differentially significant between the two species. This may imply that thermophiles exhibit increased control over the placement of mobile residues within proteins that bind to rRNA.

**Table 4:** ANM enrichment factors and significance for 30S proteins.

	*T. Thermophilus*		*E. Coli*	
	EF	p-value	EF	p-value
**S02**	0.97	0.505	1.05	0.352
**S03**	2.22	0.001	2.07	<0.001
**S04**	1.39	0.013	1.44	0.005
**S05**	1.78	0.004	1.62	0.012
**S06**	0.83	0.452	1.11	0.278
**S07**	2.76	<0.001	1.85	0.041
**S08**	1.35	0.074	1.27	0.108
**S09**	2.15	<0.001	1.73	<0.001
**S10**	1.40	0.010	1.73	<0.001
**S11**	1.69	0.008	3.20	<0.001
**S12**	1.58	0.001	1.40	0.007
**S13**	1.43	0.002	1.48	<0.001
**S14**	1.55	0.017	1.10	0.179
**S15**	1.36	0.005	1.23	0.038
**S16**	1.67	0.004	1.02	0.428
**S17**	1.48	0.005	1.33	0.063
**S18**	0.58	0.936	0.93	0.585
**S19**	1.09	0.125	0.88	0.166
**S20**	1.33	0.031	1.24	0.063

Note: EF is the enrichment factor, defined as the ratio of root mean square fluctuations for contacting over non-contacting residues. The P-value is the statistical significance computed with a permutation test. See text for details.

### Conclusion

Several important conclusions can be reached based on the above reported results. First, the positively charged residues on r-proteins must play important roles in binding with 16S rRNA, as noted earlier [10], [46]–[47]. A significantly higher percentage of contacts between r-proteins and rRNA are formed by these positively charged and hydrogen bonding residues. We also see that r-proteins from a thermophilic species (*T. thermophilus*) have more positively charged residues than a mesophilic species (*E. coli*), which correlates with the fact that thermophilic ribosomes must maintain stronger (or a larger number of) interactions in order to function at considerably higher temperatures. Second, as previously discussed [36], conformational changes of r-proteins could take place during 16S rRNA binding. Our study clearly shows that α-helix 1 of S20 is unstable in solution by itself and exhibits large conformational changes. In contrast, S15 and S17 adopt stable conformations in solution, which agrees with the earlier suggestion [37] that ribosomal proteins do not undergo structural changes during assembly. We attribute the differences in these behaviors to the extent of solvent exposure the protein experiences within the assembled subunit. In the ribosome, S15 and S17 are primarily solvent exposed so their solution structures would be likely to more closely resemble their bound structures, whereas S20 is deeply embedded in the 16S RNA, and its association with its RNA binding site stabilizes the flexible portion of α-helix 1. Third, analyses of residue mobilities reveal that RMSF values for contact residues are statistically higher than those for other residues. This means that contacting regions are more enriched with mobile residues than non-contacting regions, which supports previous observations [37] that the flexible regions of ribosomal proteins are usually the locations of RNA contacts. However, this does not mean that all contact residues are located in the flexible loop regions. It is important to point out that there are many contact residues found in α-helices and β-sheets that exhibit low to moderate RMSF values. The trend that contact residues being enriched with mobile residues holds for most of 30S r-proteins, with only a few distinct exceptions like S2, S6, S18. Their exceptions however could be traced to peculiar known facts such as dimerization between S6 and S18. The increased mobility of contact residues could ensure more efficient binding and even aid in the binding site preparation for later binding proteins by actively associating with their 16S binding partners and helping to fold and maintain the appropriate rRNA tertiary structure. The *T. thermophilus* exhibited higher enrichment factors than the *E. Coli*, which may point to a novel adaptation of thermophiles – the increased control over the placement of highly mobile residues.

## Methods

### Analysis of contacts in the assembled 30S subunits

In the current study, we analyze the crystal structures of the 30S subunits from the *Escherichia coli* (PDB [64] ID 2AVY [9]) and *Thermus thermophilus* ribosomes (PDB ID 1J5E [10]). Structural and sequence alignments of r-proteins found in the two species were done with Molecular Operating Environment (MOE) software (Chemical Computing Group). Contacts between r-proteins and 16S rRNA were analyzed using our own computer program. A contact point was defined as any atom of a protein residue found within a 3.5 Å cut-off distance from any 16S nucleotide atom. That amino acid was labeled as a “contact” residue. The total number of “contacts” between one r-protein and the 16S rRNA may exceed the total number of contacting residues identified in the protein because an amino acid may be within cutoff distance of more than one nucleotide, thus counting as more than one contact. The identity and position of these contact residues found in the assembled 30S subunit were recorded and used for further analysis.

### Molecular Dynamics simulations

Molecular dynamics (MD) simulations were run using the AMBER 10 software package [65] and the parmbsc0 force field [66], an optimization of the Amber99 force field for nucleic acids and proteins. The starting conformations of r-proteins for the MD were obtained from the crystal structures of the 30S subunits (*E. coli* 2AVY and *T. thermophilus* 1J5E). Counterions were added to neutralize the charge of the protein, and an additional 10 potassium and 10 chloride ions were added to create a low salt concentration. The protein systems were then solvated using a rectangular box of TIP3P water [67]. The systems were subjected to two minimization cycles: 1000 steps with the protein fixed and 5000 steps unrestrained. Afterward, a 100 ps warm-up MD simulation was run at constant volume by increasing temperature from 0 to 300 K, with the protein fixed using a restraint constant of 10.0 kcal·mol^−1^·Å^−2^. The MD simulation then switched to the NPT ensemble (p = 1.0 bar), using the Langevin thermostat with a collision frequency of 1.0 ps^−1^, to equilibrate the ions and water density for 2 ns. The restraint force on the protein was then removed and the production run began with the NPT ensemble (p = 1.0 bar) using a time step of 2 fs. All simulations used the SHAKE algorithm [68]–[69] to constrain covalently bonded hydrogen atoms and the Particle Mesh Ewald (PME) method [70] to calculate long-range electrostatic interactions, with a cutoff distance of 10.0 Å. Histidines are represented as HIE (neutral charge: hydrogenated N^ε^, aromatic N^δ^). Duplicate MD simulations were performed to verify that the reported dynamic behaviors of each protein are representative in the final MD runs. MD production runs were performed for at least 200 ns, which should be of sufficient length to establish the conformational stabilities of proteins of this size.

Using Ptraj to monitor the overall structural changes in reference to the starting structure, the root-mean-square deviation (RMSD) for each protein was calculated as a function of production run time. If the plot of the RMSD versus time forms a plateau, the protein likely adopts a solution-stable conformation; however, a widely fluctuating RMSD plot indicates a flexible protein in solution. To quantify the mobility of each residue, root-mean-square fluctuations (RMSF) were calculated using the average protein conformation as the reference state. The RMSF values presented in this paper are calculated from 10 ns to the end of each simulation (approximately 200 ns) to allow adequate time for the protein to fully adopt its stable solvated conformation, if one was at all achieved. This ensures that the RMSF plot differentiates flexible residues from stationary residues during the time that the protein samples its solution-stable conformations. In both RMSD and RMSF calculations, all atoms were included.

The RMSF is related to the experimental B-factors reported by crystallographers, through a simple relationship (B-factor = (8/3)π^2^(RMSF)^2^), which could be compared with the experimental measured B-factors reported in the PDB files of the 30S subunits. However, the experimental B-factors for each r-protein found in the 30S subunits were nearly featureless for individual proteins, probably because the reported B factors reflect the mobility of the atoms within the whole assembled subunit and are not representative of the individual r-proteins. Hence, we did not compare the B-factors calculated from MD simulations with the experimental B-factors.

Snapshots of each protein at various stages throughout the simulations were visualized using Visual Molecular Dynamics [71] (VMD) to identify the flexible and stable regions of the protein. All images were made with VMD, which is developed with NIH support by the Theoretical and Computational Biophysics group at the Beckman Institute, University of Illinois at Urbana-Champaign.

### Elastic network modeling

Because the Molecular Dynamics simulations require significant resources, we have also chosen to model the dynamics of the complete set of 30S ribosomal proteins with the more computationally efficient elastic network model [72], using the Anisotropic Network Model in particular [63], [73], ANM models permit us to investigate the dynamics of all of the 30S proteins more quickly but with less detail in the observed dynamics than MD, but with greater overall certainty about the large-scale motions of the structures. ANM models are constructed using the crystallographic C^α^ coordinates of each protein and a cutoff of 13 Å. Due to its coarse-grained design, the ANM is subject to the “tip effect” [74]–[75] in which highly extended points (C^α^) experience exaggerated motions, which would place disproportionate weight on the most mobile residues. To compensate for this effect, we calculate the RMSF of each residue position in each structure and remove extreme outliers from subsequent analyses. The “tip effect” residues removed in this study are Arg88 and Gly89 from *T. thermophilus* S15, and Gly8, Val9, Val10, and Val11 from *T. thermophilus* S17. We also use RMSF to make comparisons between 16S rRNA contacting residues and non-contacting or highly conserved residues. The definition of contacting residues and conserved residues is the same in both the ANM calculations and the MD studies.

### Statistical analysis of contact residue mobility

To statistically determine linkages between highly mobile and contacting residues or conserved residues from both ANM calculation and MD simulation, we calculate an enrichment factor for each protein defined as the ratio of the average RMSF for contacting over non-contacting residues. An enrichment factor greater than 1 implies that the contacting residues are more mobile than the non-contacting residues. However, an enrichment factor less than 1 implies the reverse. The statistical significance (p-value) of the enrichment factor is calculated based on the permutation test explained as follows. For a protein of N residues, C of which are contacting, we have an observation of the enrichment of RMSF at the contacting residues relative to the non-contacting residues. Let this ratio be O. We then randomly select C residues from the protein and calculate the analogous ratio between this random set and its compliment. Performing the random selection 10,000 times, we construct a distribution of enrichment values within random sets of C residues. The significance (p-value) of our initial observation, O, is then the proportion of random samples that have an enrichment greater than O. A small p-value (e.g., p<0.01) implies that a random set of C residues is unlikely to have an enrichment factor equal or greater than the observed ratio O. This not only means that the contacting residues are more mobile than the non-contacting residues, but that there are very few subsets of size C exhibiting the same magnitude of mobility.

## Supporting Information

Table S1 **S15 Contact residues, at 3.5 Å cutoff distance.** This table provides the protein residues of S15 in contact with 16S RNA, for both *E. coli* and *T. thermophilus*. Residues colored red have conserved identity in the sequence alignment of the two proteins; those in green have conserved type, i.e. basic, acidic, polar, nonpolar, or aromatic. Some residues may contact more than one nucleotide.
(DOC)

Table S2 **S17 Contact residues, at 3.5 Å cutoff distance.** This table provides the protein residues of S17 in contact with 16S RNA, for both *E. coli* and *T. thermophilus*. Residues colored red have conserved identity in the sequence alignment of the two proteins; those in green have conserved type, i.e. basic, acidic, polar, nonpolar, or aromatic. Some residues may contact more than one nucleotide.
(DOCX)

Table S3 **S20 Contact residues, at 3.5 Å cutoff distance.** This table provides the protein residues of S20 in contact with 16S RNA, for both *E. coli* and *T. thermophilus*. Residues colored red have conserved identity in the sequence alignment of the two proteins; those in green have conserved type, i.e. basic, acidic, polar, nonpolar, or aromatic. Some residues may contact more than one nucleotide.
(DOCX)
