# Supplementary material for: A Computational Investigation on the Connection between Dynamics Properties of Ribosomal Proteins and Ribosome Assembly
Source: PLoS Comput Biol. 2012 May 24;8(5):e1002530. doi: 10.1371/journal.pcbi.1002530 (PMC3359968; doi:10.1371/journal.pcbi.1002530)
Supplement: Table S2 — S17 Contact residues, at 3.5 Å cutoff distance. This table provides the protein residues of S17 in contact with 16S RNA, for both E. coli and T. thermophilus. Residues colored red have conserved identity in the sequence alignment of the two proteins; those in green have conserved type, i.e. basic, acidic, polar, nonpolar, or aromatic. Some residues may contact more than one nucleotide. (DOCX) [file pcbi.1002530.s002.docx]

**Linkage between Dynamics and Assembly of Ribosomal Proteins**

Brittany Burton, Michael T. Zimmermann, Robert L. Jernigan and Yongmei Wang

**Supplementary Data**

| ***Table S2: S17 contact residues; 3.5Å cutoff distance*** | | | | | | | | | |
| --- | --- | --- | --- | --- | --- | --- | --- | --- | --- |
| ***E. coli* (21)** | Ile5 | Met17 | Ser20 | Phe37 | Lys43 | Arg65 | Ser68 |  |  |
|  | Ser14 | Glu18 | Ile33 | Arg40 | Leu44 | Pro66 | Lys69 |  |  |
|  | Lys16 | Lys19 | Tyr34 | Thr41 | His45 | Leu67 | Lys71 |  |  |
| ***T. thermophilus* (36)** | Pro2 | Gln16 | Tyr32 | Lys40 | Ser62 | Ser66 | Arg70 | Tyr95 | Gly102 |
|  | Lys3 | Lys17 | Lys34 | Lys41 | Arg63 | Lys67 | Arg72 | Leu98 | Gly103 |
|  | Ser12 | Thr18 | Arg38 | Tyr42 | Pro64 | Arg68 | Arg91 | Ser99 | Lys104 |
|  | Met15 | Arg25 | Ser39 | Glu61 | Ile65 | Lys69 | Asn94 | Lys100 | Ala105 |

Note: Residues colored red have conserved identity in the sequence alignment of the two proteins; those in green have conserved type, i.e. basic, acidic, polar, nonpolar, or aromatic. Some residues may contact more than one nucleotide.
